# Orchidopexy for Ascending Testicles in Adulthood After Remote Hernia Repair: A Report of Two Cases

**DOI:** 10.7759/cureus.39885

**Published:** 2023-06-02

**Authors:** Moshe Wald

**Affiliations:** 1 Urology, University of Iowa Hospitals and Clinics, Iowa City, USA

**Keywords:** orchiopexy, adults, inguinal hernia repair, ascending testicle, orchidopexy

## Abstract

Testicular ascent to the inguinal region after hernia repair has been previously reported as a rare complication of this surgery in pediatric patients. This article presents two cases of adult patients with ascending testicles after inguinal hernia repair that was performed in childhood. Both men underwent orchidopexy through a combined inguinal and scrotal approach, the latter for the creation of a sub-dartos pouch. In both cases, this intervention was completed successfully without complication and resulted in a satisfactory post-operative position of the testicles in the scrotal sac. This surgical approach appears to be a safe management option for adult men with ascending testicles after inguinal hernia repair.

## Introduction

The development of ascending testicles after inguinal hernia repair has been reported in children [1,2]. Separate from the iatrogenic failure to replace the testes in the scrotum during pediatric hernia repair, the term “ascending testes” was used to describe the spontaneous migration of the testicles to the inguinal area after being placed in the scrotum during pediatric hernia repair [1].

Ascending testis has been reported to be a rare occurrence after repair of an inguinal hernia in children, ranging from 0.058% to 0.43% [1,2]. While data regarding ascending testicles after inguinal hernia repair have been available from the pediatric literature, the occurrence of this condition in adult patients has not been reported.

This article presents two cases of adult patients who underwent surgical intervention for ascending testicles, describes the surgical technique that was used, and provides the surgical outcomes.

## Case presentation

Case 1

The first patient is a 26-year-old male, who presented to our clinic regarding a left undescended testicle. He underwent bilateral hernia surgery in infancy. He then had an orchiopexy 11 years ago in another country, as his left testicle ascended to the inguinal region following the hernia repair. The patient never felt the testicle in the scrotum after that attempt at orchiopexy but felt it in the inguinal area. He has not felt any masses. He reported occasional pain in the left inguinal region and up into the left lower abdomen, for which he requested treatment. He had no chronic illnesses. Physical examination revealed the left testicle to be palpable in the inguinal region; the right testicle was descended into the scrotum, of normal size, and without palpable masses.

**Figure 1:**
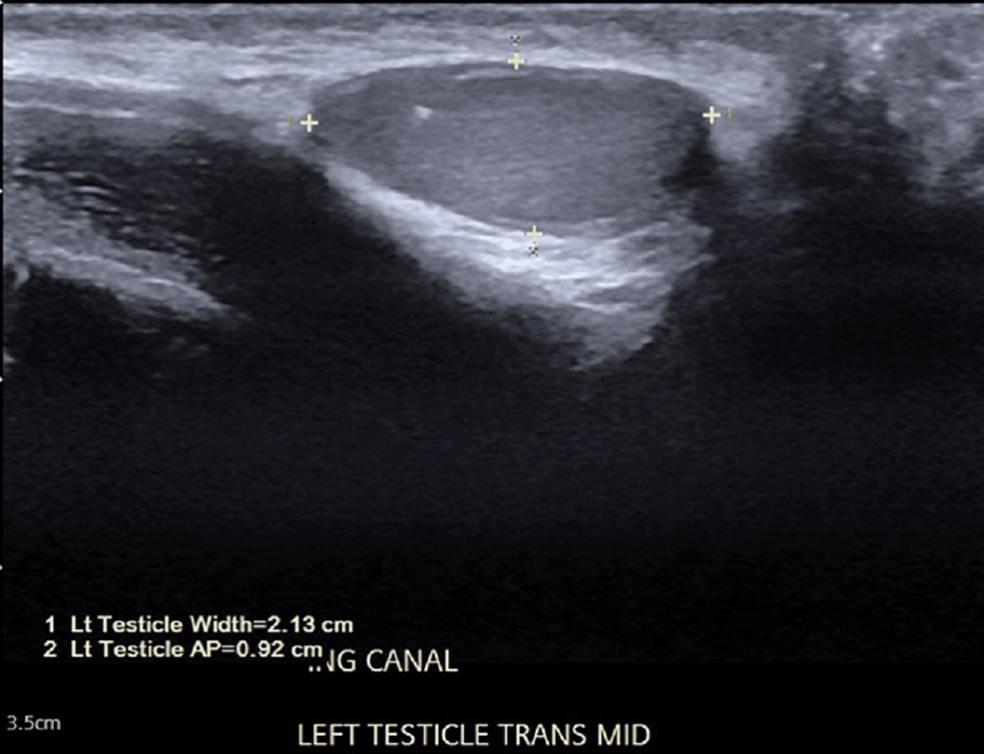
Pre-operative ultrasound of the patient in case 1 Ultrasound of the patient in case 1 two years ago, which demonstrated an asymmetric small size and mildly hypoechoic left testicle; the right testicle was descended and normal.

Ultrasound performed five years ago confirmed left testicle positioned in the left inguinal canal, mildly atrophic without masses. Repeat ultrasound two years ago demonstrated an asymmetric small size and mildly hypoechoic left testicle; the right testicle was descended and normal (Figure 1).

After counseling, the patient elected to proceed with left orchidopexy, which was performed under general anesthesia. The left testicle was then brought down to the scrotum and anchored in a sub-dartos pouch that was created in the left hemiscrotum. The patient tolerated the procedure well and was transferred to the recovery unit in stable condition.

The patient recovered well from surgery with no complication. At follow-up 17 months after surgery, he reported complete resolution of pre-operative symptoms. His left testicle was palpable within the upper left hemiscrotum with no palpable masses.

Case 2

The second patient is a 37-year-old male, who initially presented to our clinic seven years ago regarding infertility. His past medical history was unremarkable. He reported a history of left inguinal hernia repair when he was three years old.

Physical examination revealed descended right testicle of normal size and consistency and no varicocele. The left testicle was noted in the inguinal canal, which limited its evaluation. He had three semen analyses seven years ago, which showed severe oligospermia (sperm concentration ranged from 0.008 to 0.03 million/mL [reference range: 15 million/mL and above]) and necrospermia. Hormonal evaluation revealed serum levels of follicle-stimulating hormone (FSH) level at 9.5 mIU/mL (reference range: 1.4-18.1 mIU/mL), luteinizing hormone (LH) at 4.3 mIU/mL (reference range: 1.5-9.3 mIU/mL), prolactin at 4.0 ng/mL (reference range: 2.1-17.7 ng/mL), and testosterone at 330 ng/dL (reference range: 249-836 ng/dL). Karyotype was 46,XY, and Y chromosome microdeletion assay was negative. Scrotal ultrasound seven years ago showed a normal right testicle in the scrotum, with the left testicle in the inguinal canal.

The patient was started on clomiphene citrate, and his serum testosterone increased to 480 ng/dL. However, semen analysis three months later showed normal volume azoospermia. He underwent right testicular sperm extraction seven years ago, during which mature and viable sperm was successfully retrieved and was cryopreserved. He returned to our clinic a year ago regarding his left undescended testicle. He then reported that he and his wife have a 10-month-old boy, who was conceived through in vitro fertilization.

**Figure 2:**
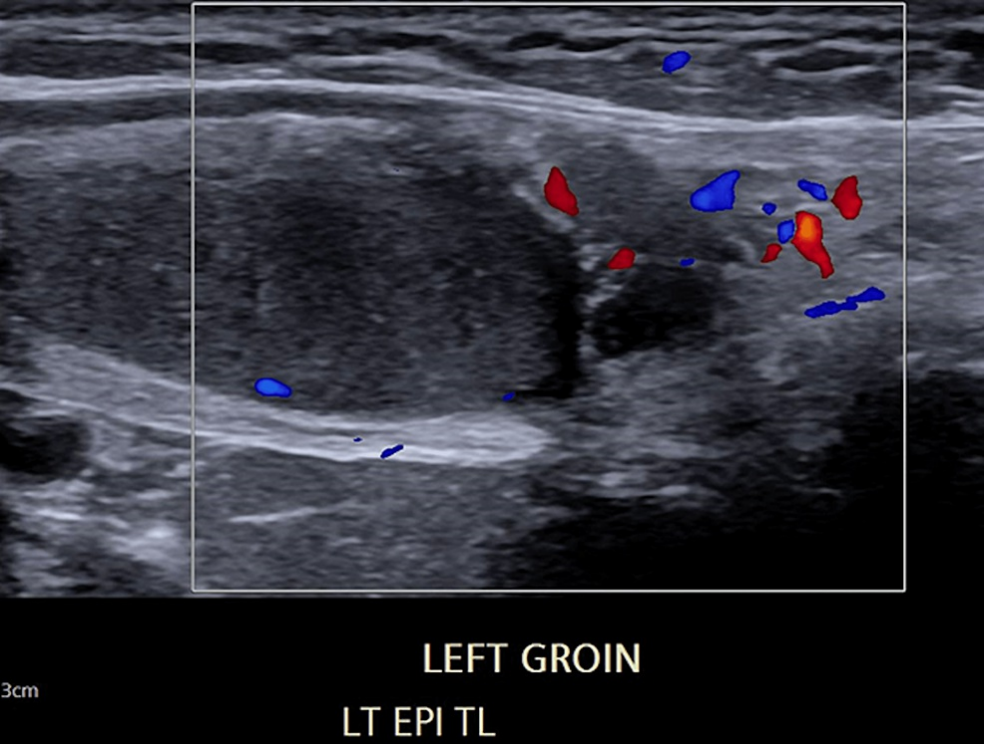
Pre-operative ultrasound of the patient in case 2 Ultrasound of the patient in case 2 a year ago, which showed normal size (4.5 x 2.7 x 1.7 cm), shape, and echotexture of the right testicle, with scattered microliths; the left testicle was in the left groin, smaller in size (3.6 x 2.0 x 1.5 cm), and normal in shape and echotexture, with no focal lesions.

No surveillance has been performed since he was last seen in our clinic six years ago. He denied any pain in the left testicle. While he was able to bring the testicle down into the very high scrotum, this was difficult to do and did not allow for a reliable examination of the left testicle. Repeat scrotal ultrasound a year ago showed normal size (4.5 x 2.7 x 1.7 cm), shape, and echotexture of the right testicle, with scattered microliths; the left testicle was located in the left groin, smaller in size (3.6 x 2.0 x 1.5 cm), and normal in shape and echotexture, with no focal lesions. Color Doppler was normal in both testicles (Figure 2).

After appropriate counseling, the patient elected to proceed with left orchidopexy in an attempt to bring the testicle to a palpable position, allowing for self-examinations. This surgery was performed under general anesthesia. The left testicle was brought down into the left hemiscrotum, where it was anchored in the sub-dartos pouch. This resulted in the left testicle in the dependent portion of the left hemiscrotum, approximately symmetric to the contralateral side. The patient tolerated the procedure well with no complications and was discharged home on the day of surgery.

The patient was seen in the clinic six weeks after surgery, at which time he was doing well and without any pain. Surgical incisions were healed, and both testicles were descended and well within the scrotal sac.

## Discussion

The development of ascending testicles after inguinal hernia repair has been reported in the pediatric literature and is considered a rare post-operative complication in this age group, with its occurrence ranging from 0.058% to 0.43% [1,2]. One of the possible mechanisms that were suggested for this condition was an iatrogenic failure to replace the testes in the scrotum during pediatric hernia repair. However, cases of spontaneous migration of the testicles to the inguinal area after being placed in the scrotum during pediatric hernia repair have been reported and considered ascending testes [1]. The development of adhesions of the spermatic cord after inguinal hernia repair was suggested as a possible mechanism for ascending testes.

In a large series of 910 boys who underwent inguinal hernia repair, ascending testes were detected in four (0.43%) of the patients, who were 1-3 years old [1]. Human chorionic gonadotropin (hCG) was suggested as a possible treatment in the early post-operative period for pediatric patients, as it led to improvement in two of these patients, but it was concluded that orchidopexy may be needed later.

This article presents two cases of adult patients with unilateral undescended testicles that developed after inguinal hernia repair earlier in life. The first patient had a failed attempt at orchidopexy 11 years ago. Ascending testicle after an inguinal orchidopexy was reported to range from 0.2% to 13% [3]. However, in the absence of medical records regarding the previous attempt at orchidopexy (which was performed in another country), it would be difficult to discuss the reasons for failure of that procedure.

In both cases, patients’ decision to proceed with surgical repair of this condition was driven by their desire to have the testicle in a position that would allow for routine self-examinations, taking into consideration that extra-scrotal testicular location is associated with a considerable risk for the development of testicular cancer [4,5]. The first patient also had occasional left inguinal and lower abdominal pain, for which he requested treatment.

Both men underwent orchidopexy through a combined inguinal and scrotal approach, with the latter for the creation of a sub-dartos pouch. Of note, the first patient underwent a failed attempt at an orchiopexy 11 years ago in another country. The patient never felt the testicle in the scrotum after that surgery but felt it in the inguinal area. In both cases, this intervention was performed on an outpatient basis, was completed successfully without complication and resulted in a satisfactory post-operative position of the testicles in the scrotal sac.

This surgical approach appears to be a safe and feasible management option for adult men with undescended testicles that developed after previous inguinal hernia repair. This procedure would allow future self-testicular examinations and may lead to resolution of discomfort if present and associated with the extra-scrotal location of the testicle.

## Conclusions

Testicular ascent to the inguinal region after hernia repair is a rare occurrence, which thus far has been reported only in pediatric patients. This article presents two cases of adult patients with ascending testicles after inguinal hernia repair. Ascending testicles in adult patients who underwent inguinal hernia repair could be successfully managed by orchidopexy, performed through a combined inguinal and scrotal approach, with the latter for the creation of a sub-dartos pouch.
